# Rotavirus vaccine protection in low-income and middle-income countries

**DOI:** 10.1016/S1473-3099(19)30263-4

**Published:** 2019-06-06

**Authors:** Girija Ramakrishnan, Jennie Z Ma, Rashidul Haque, William A Petri

**Affiliations:** University of Virginia, Charlottesville, VA 22908, USA (GR, JZM, WAPJr); and International Centre for Diarrhoeal Disease Research, Dhaka, Bangladesh (RH)

Global under-5 mortality decreased by 50% between 1990 and 2016. The UN Sustainable Development Goals (SDGs) call for under-5 mortality to be no greater than 25 deaths per 1000 livebirths. Since 1990, there has been tremendous progress towards this goal, with a reduction from 142 deaths per 1000 livebirths to 65 deaths per 1000 livebirths for the poorest quintile in low-income and middle-income countries (LMICs).^1–2^ Diarrhoeal diseases are estimated to result in 424 000 deaths in children younger than 5 years in 2017, making them the fourth leading cause of death after prematurity, birth trauma, and pneumonia.^3^ Control of diarrhoeal diseases is complicated by the fact that there are ten different pathogens that together are responsible for the vast majority of cases.^4^ Of these ten causes of diarrhoea, for only one, rotavirus, is there a vaccine.

A further problem with control of diarrhoeal diseases is that the oral rotavirus vaccine is less effective in LMICs.^5,6^ In our work,^6^ we found protection efficacy against severe rotavirus diarrhoea in Bangladesh was 74%, and that rotavirus vaccine failure was associated with a chronic inflammatory intestinal disease called environmental enteric dysfunction (EED). EED was present in 80% of infants aged 12 weeks in an urban slum of Dhaka, Bangladesh, as measured by faecal biomarkers of inflammation.^7^ EED was associated with oral vaccine failure but not failure of systemically administered vaccines, such as the diphtheria, tetanus, and pertussis vaccine. We measured rotavirus vaccine failure as the occurrence of rotavirus-caused diarrhoea in a vaccinated child. Biomarkers of EED and maternal health accounted for 24% of rotavirus vaccine failures.^7^

One cause of EED is enterovirus infection. We found that enterovirus in stool on the day of vaccination was associated with diminished rotavirus IgA vaccine response.^8^ It is probable that rotavirus vaccine failure is in part due to a failure of vaccine virus replication resulting from an active gut antiviral immune response.^8^

Despite the issue of a less effective response in LMICs, the WHO Strategic Advisory Group of Experts on Immunization has recommended rotavirus vaccination as part of the Expanded Program on Immunization for all regions of the world. This recommendation is in large part because of the benefit in reduction in child mortality and morbidity from even a partly effective vaccine.

In *The Lancet Infectious Diseases,* Andrew Clark and colleagues^9^ examined all randomised controlled trials of rotavirus vaccination published before April 4, 2018. They classified the trials’ settings into low-mortality, medium-mortality, and high-mortality strata, and estimated stratum-specific vaccine efficacy over time from a Bayesian hierarchical Poisson meta-regression model, which accounted for observed study-level characteristics and unmeasured heterogeneity among the studies. Clark and colleagues^9^ also converted the cumulative vaccine efficacy to instantaneous vaccine efficacy to account for the potential temporal changes of rotavirus failure risk and tested for waning efficacy over the first year of life. The result was a validation of low rotavirus vaccine efficacy in low-income and high-mortality settings for the prevention of severe rotavirus diarrhoea, with only 44% effectiveness (95% credibility interval 27–59) after 12 months compared with 94% (87–98) effective in high-income and low-mortality settings. Vaccine efficacy waned more rapidly in high-mortality settings leading to the important observation that improvements in efficacy need to address both instantaneous and prolonged immunity.

The research questions arising from this Article are centred on how to improve vaccine efficacy. Rotavirus vaccination expansion throughout high-mortality countries should help to meet the SDG goals, and even small improvements in vaccine efficacy will have a measurable effect on infant mortality due to diarrhoeal diseases. Interference from EED and enterovirus suggests that the gut metabolome and microbiome could have a role, and any metabolites found to be associated with vaccine success might hold potential as vaccine adjuvants. Avoidance of interfering enterovirus infection and EED by administering the first dose of the vaccine at birth is another potentially beneficial strategy.

A promising alternative strategy is to move from a live virus oral vaccination to an inactivated vaccine administered intramuscularly or intradermally, either alone or in combination with oral vaccine, as borne out by experience with the poliovirus vaccination.^10^ A heat-inactivated parenteral vaccine can induce mucosal immunity in animal models,^11–13^ and a parenteral subunit vaccine has been shown to be safe and efficacious in a clinical trial with infants in South Africa,^14^ spurring a phase 1 and 2 trial that is underway. Indeed, the ability to effectively induce mucosal immunity using parenterally administered vaccines with select adjuvants might render the oral rotavirus vaccine unnecessary in the not-too-distant future.^15^
